# Assessment of the Antimicrobial Effect of Momordica charantia (Bitter Gourd Oil) on Periodontal Pathogens: An In Vitro Study

**DOI:** 10.7759/cureus.75209

**Published:** 2024-12-06

**Authors:** Pranavi Jayaraj, Hemalatha Ramakrishnan, Surya Paulraj, Priyadarshini Govindarajan, Haritha Muralidharan, Manasa Prabakar

**Affiliations:** 1 Department of Periodontology, Karpaga Vinayaga Institute of Dental Sciences, Chengalpet, IND

**Keywords:** bitter gourd, diabetes mellitus, minimum inhibitory concentration(mic), momordica charantia, periodontitis, phytochemical analysis

## Abstract

Background

Chronic periodontitis is primarily caused by various bacterial species present in the plaque biofilm, which trigger a host inflammatory response. This leads to the abnormal release of inflammatory mediators such as proinflammatory cytokines (interleukin-1, interleukin-6, interleukin-8, and tumor necrosis factor-α), which are free radicals that cause alveolar bone resorption and tooth loss. ​​​*Momordica charantia *(bitter gourd) is a widely used medicinal plant for the treatment of numerous diseases such as skin infections, diabetes, metabolic disorders, and carcinomas for several decades. *Momordica charantia *modulates the host immune response and inhibits the formation of proinflammatory mediators, thereby preventing periodontal tissue destruction.

Aim

This study aims to assess the antimicrobial activity of bitter gourd oil against early colonizing microorganisms of chronic periodontitis.

Materials and methods

In this in vitro study, plaque samples were obtained from 10 subjects with chronic periodontitis. *Streptococcus pyogenes* were subcultured from plaque samples, and the minimum inhibitory concentration (MIC) of bitter gourd oil was determined using the disc diffusion method. The presence of alkaloids, terpenoids, quinones, flavonoids, and phenols in *Momordica charantia* (bitter gourd oil) was determined by phytochemical testing. Statistical analysis was conducted using the ANOVA test.

Results

Phytochemical analysis identified alkaloids, terpenoids, flavonoids, and phenols in the oil sample. The higher concentrations of *Momordica charantia *oilexhibited larger zones of inhibition against *Streptococcus pyogenes.*

Conclusion

*Momordica charantia* demonstrates antibacterial properties against *Streptococcus pyogenes*, making it a potential therapy for periodontal disease. Due to the setbacks of various antimicrobial agents such as drug interactions and antimicrobial resistance, medicinal plants such as *Momordica charantia* can serve as an alternative and desirable choice of treatment in patients with chronic periodontitis.

## Introduction

Chronic periodontitis is one of the most common oral diseases that affects those between 30 and 60 years of age. It is a polymicrobial disease that initially manifests as gingivitis and slowly progresses to the destruction of periodontal ligament and alveolar bone, and eventually leads to tooth loss. This condition is aggravated by exacerbation of the host immune response, which leads to the abnormal release of inflammatory mediators and tissue destruction products. The tooth-associated plaque is predominated by gram-positive rods and cocci, while an increased prevalence of gram-negative bacteria and spirochetes is seen in gingival crevice-associated plaque. Oral commensal Streptococcus species are found to be present in saliva and subgingival plaque samples of patients with periodontal disease. These species contribute to periodontal tissue destruction by interacting with other pathogenic microorganisms in the plaque biofilm. The goal of periodontal therapy is to reduce dental plaque formation and eliminate the disease-causing microorganisms, thereby improving periodontal health. Various antimicrobial agents have been widely used as an adjunct to conventional periodontal therapy [1]. Antimicrobial agents used for the treatment of periodontitis, such as amoxicillin, tetracyclines, and metronidazole, target specific groups of microorganisms and may not be effective in eliminating the entire microflora, which exhibits synergism in periodontitis. Though antibiotics have a wide spectrum of activity against periopathogens, some of the setbacks encountered are hypersensitivity, allergic reactions, and antimicrobial resistance developed by various microorganisms [2]. Studies show that around 74.2% of chronic periodontitis patients showed resistance to the most widely used antibiotics in dentistry [3]. Due to the side effects of antimicrobial agents and antibiotic resistance exhibited by microorganisms, medicinal plants have gained more attention in the preventive and therapeutic management of various health conditions. *Momordica charantia* (bitter gourd) belongs to the Cucurbitaceae family and has been extensively grown in Asia, India, East Africa, and South America. It has been used to cure a variety of illnesses, including skin infections and cancer, due to its antimicrobial, anti-oxidative, anti-inflammatory, immunomodulatory, and anti-carcinogenic properties [4].

Diabetes and periodontitis have a strong bidirectional relationship and periodontitis is considered to be the sixth complication of diabetes mellitus [5,6]. The release of advanced glycation end-products (AGEs) and their interaction with the host cells result in excessive periodontal tissue breakdown and impaired wound healing. In periodontitis, the release of proinflammatory cytokines inhibits tyrosine phosphorylation of insulin receptors, which leads to insulin resistance and elevated blood glucose levels. Diabetes also exhibits concordant comorbidities such as cardiovascular disease, hypertension, obesity, hyperlipidemia, chronic vascular disease (CVD), diabetic neuropathy, and chronic kidney disease. Bioactive compounds such as Charantin, polypeptide-p, and vicine exhibit anti-diabetic and hypoglycemic effects by improving insulin secretion and regulating glucose uptake by the body [7]. *Momordica charantia* can be used as a potential choice of drug for the treatment of both diabetes and periodontitis. *Momordica charantia *has been found to possess antibacterial properties due to the existence of biochemical compounds such as alkaloids, tannins, terpenoids, and saponins [8]. This study aims to evaluate the antimicrobial properties of *Momordica charantia* by assessing the minimum inhibitory concentration (MIC) against oral pathogenic microorganisms and to analyze the phytochemicals present in *Momordica charantia* (bitter gourd oil) qualitatively.

## Materials and methods

Study design and patients

An in vitro study was conducted in the Department of Periodontology, Karpaga Vinayaga Institute of Dental Sciences, Chengalpet, Tamil Nadu, India. Plaque samples were obtained from subjects who were eligible for the study and treated with *Momordica charantia* (bitter gourd oil), amoxicillin (positive control), and hexane (negative control). The study participants were included according to the following criteria: age range between 30 and 50 years, systemically healthy individuals with probing pocket depth (PPD) ≥ 5mm, bleeding on probing, clinical attachment loss ≥ 3mm, and radiographic evidence of bone loss in more than 30% of the tooth surfaces. Individuals with any form of systemic illness or under medications, those who underwent periodontal treatment during the previous six months, smokers, and women who were pregnant and breastfeeding were excluded from the study. The sample size of the study was 10 participants. The sample size was calculated with an alpha error probability of 0.05, an effect size of 0.90, and a research power of 0.80 using the G* Power software (version 3.1.9.4). The Institutional Ethics Committee (IEC) granted approval for the study protocol and issued a certificate number (KIDS/IEC/2024/I/013).

Preparation of *Momordica charantia* (bitter gourd) oil

Dry seeds were extracted from the ripe fruits of *Momordica charantia*. The seeds were split and thoroughly rinsed with water to remove any contaminants. After being vacuum-dried, the seeds were ground into a fine powder using a milling tool. The bitter gourd oil was prepared using the Soxhlet apparatus. The powdered sample was placed in a thimble chamber, and petroleum ether was used as an extraction solvent. The chamber was heated to 60°C for 6-8 hours. The bitter gourd oil was then collected from the round bottom flask of the apparatus.

Phytochemical analysis of *Momordica charantia* oil

Phytochemical analysis was conducted to determine alkaloids, terpenoids, quinones, flavonoids, and phenols in *Momordica charantia* oil using the following biochemical tests. Yellow or creamy precipitate confirmed alkaloid compounds in the oil sample after the addition of Mayer's reagent (potassium mercuric iodide solution). Red-brown color after adding a few drops of 2% sulfuric acid confirms the presence of terpenoids in the oil sample. No color change after adding a few drops of 10% sodium hydroxide into the bitter gourd oil sample indicates the absence of quinones. Flavonoids were identified by the appearance of yellow to orange color on adding a few drops of 1% aluminum chloride into the oil sample. The presence of phenols is confirmed by the formation of a black color precipitate after adding 2mL of ferric chloride into the sample.

Plaque sample collection

**Figure 1:**
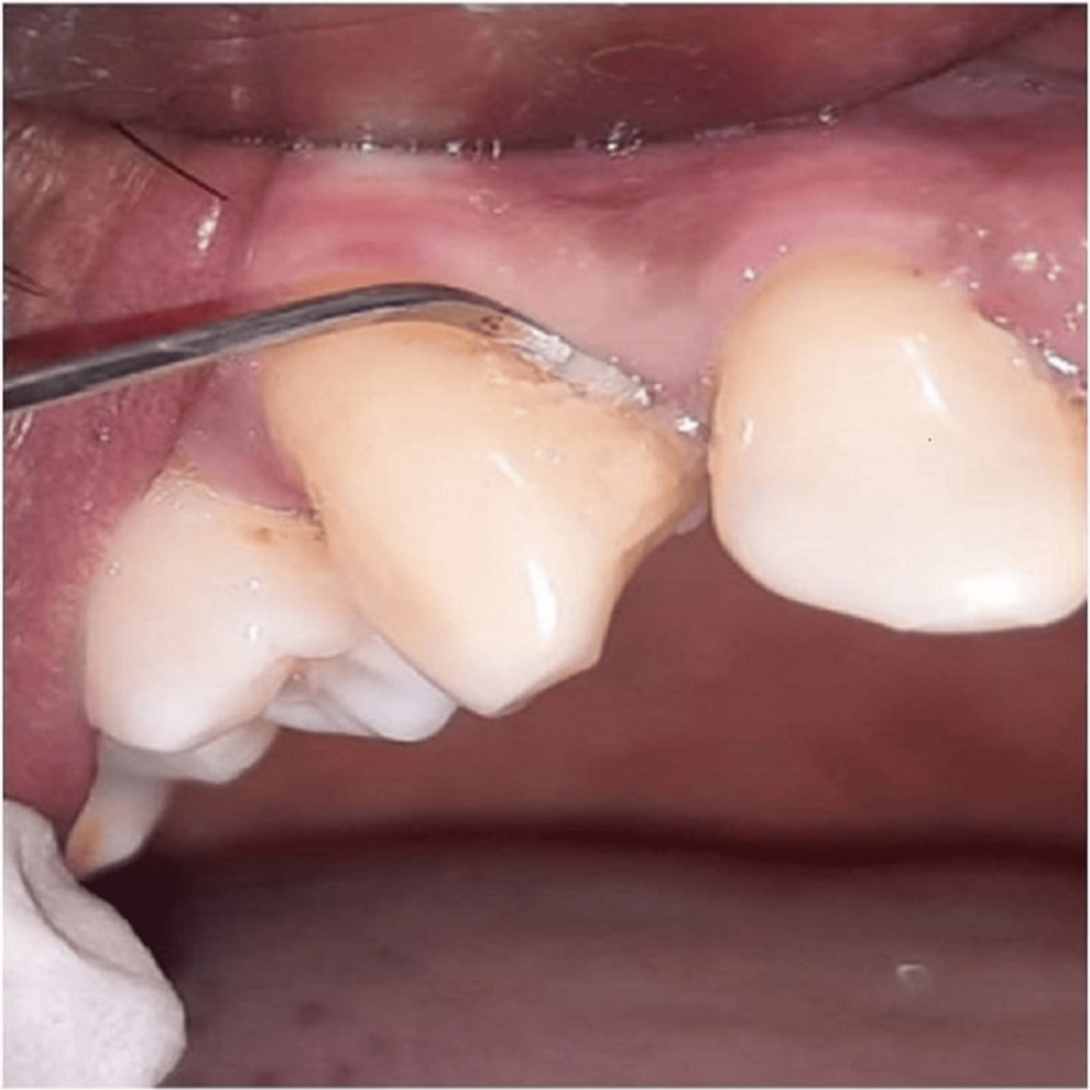
Collection of plaque sample

Following isolation with cotton rolls, plaque samples were obtained from the periodontal pocket by inserting a surgical curette (Figure 1). It was then placed in thioglycolate broth until subject to microbial analysis.

Microbial analysis of plaque sample and identification of *Streptococcus pyogenes*

Within 2 hours, plaque samples were streaked on nutrient broth-infused agar plates using a sterile inoculating loop and cultured at 37°C. After 48 hours, the colonies were identified, and plates with more than 250 colonies were taken into further microbial analysis. Bacterial colonies were then subcultured in trypticase soy agar (TSA) and incubated at 37°C for two days. A negative catalase test result confirmed the presence of *Streptococcus pyogenes*.

MIC of *Momordica charantia* oil against *Streptococcus pyogenes *using the disc diffusion method

MIC denotes the lowest concentration of any chemical or drug that can completely inhibit bacterial growth. Disc diffusion is one of the most commonly used methods for determining MIC [9]. In this method, paper discs are soaked with varying concentrations of the test sample or agents. The discs are positioned on the agar plate's surface and diffusion of the agent results in concentration gradient. The clear zone around each disc is measured to determine the least concentration of the test sample or agent required to inhibit the growth of bacteria.

**Figure 2:**
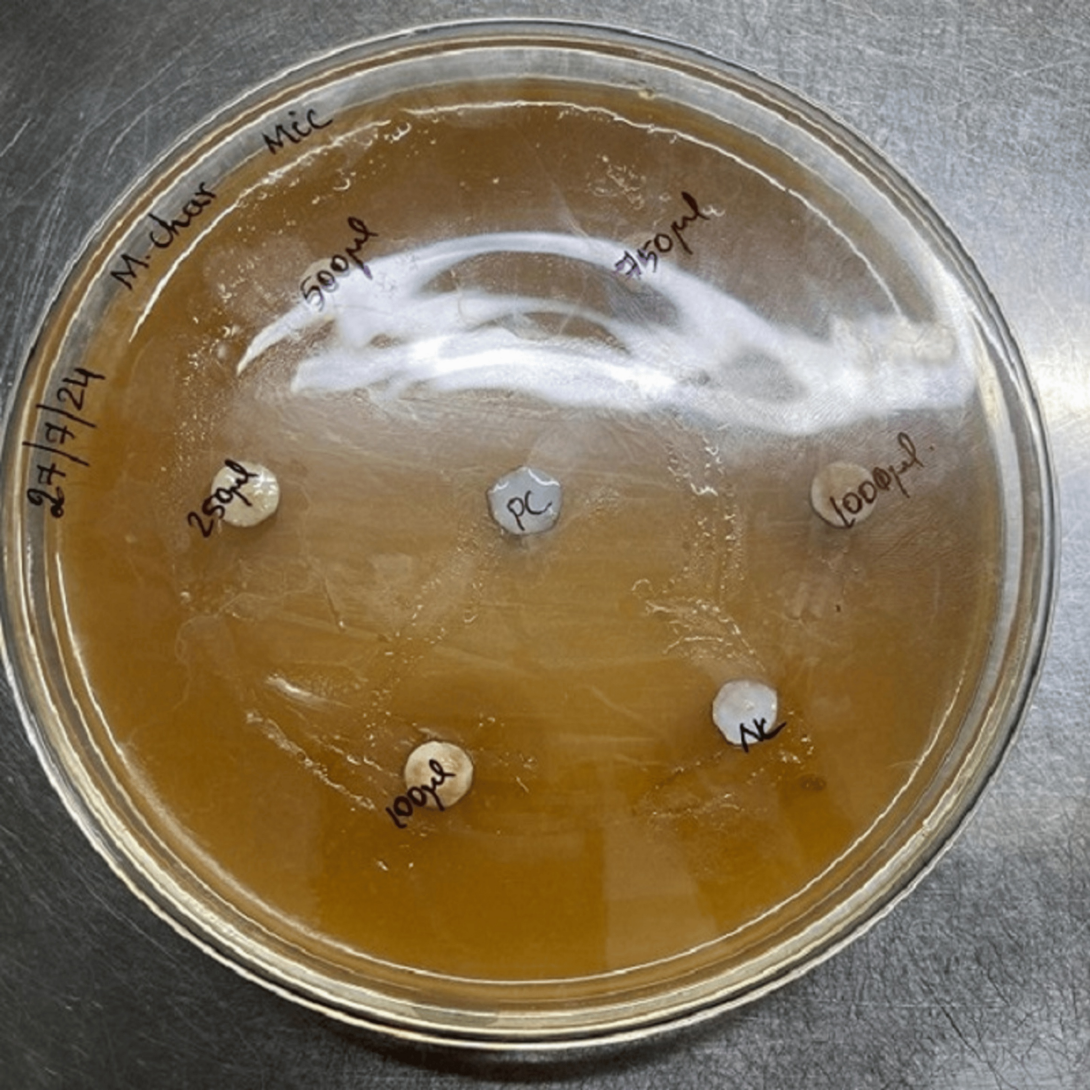
Disc diffusion method (before incubation)

**Figure 3:**
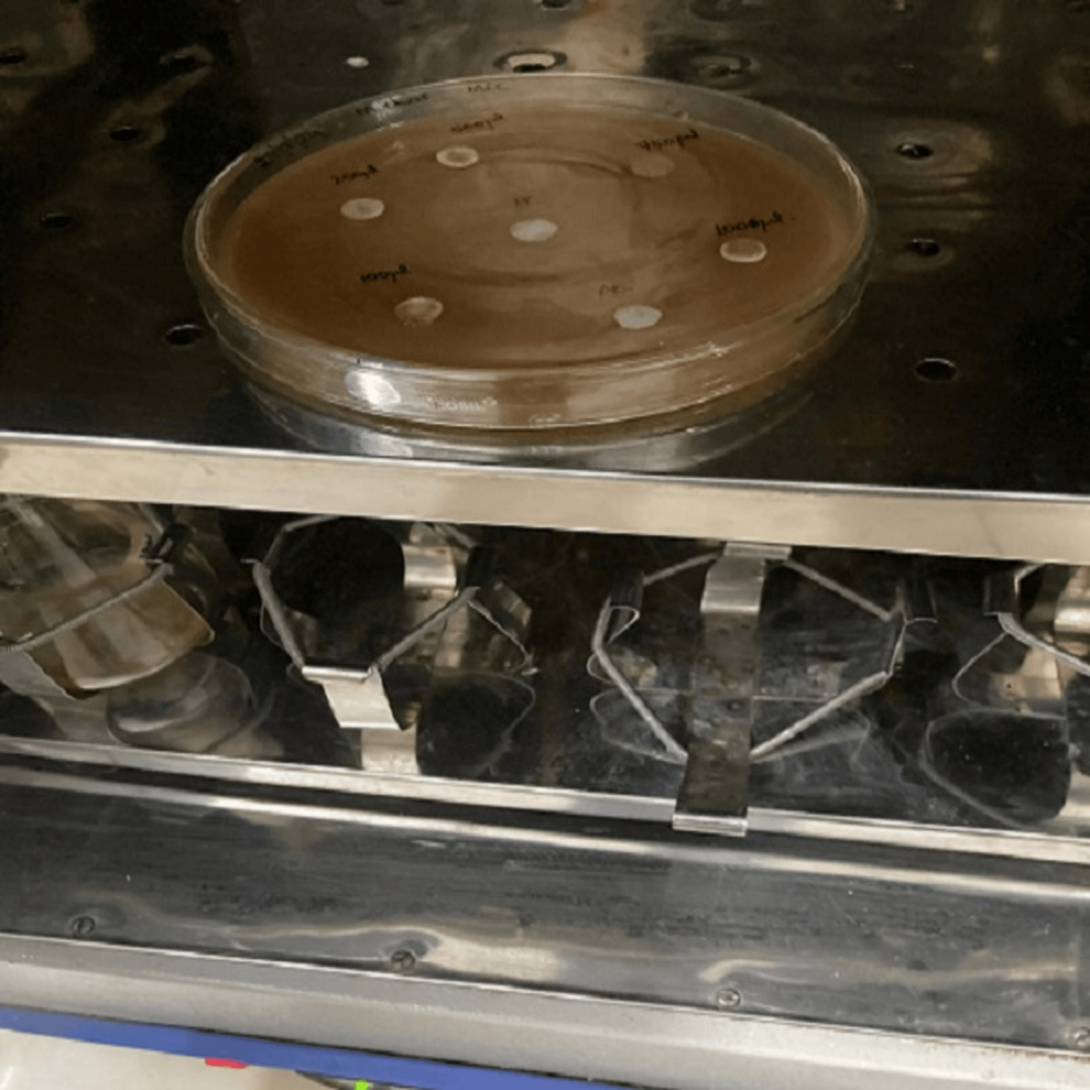
Incubation of the agar plates

In this study, the MIC of *Momordica charantia* oil was tested using the disc diffusion method. Hexane (100µL) served as the negative control, and amoxicillin (100µg/mL) served as the positive control. Paper discs were soaked in five different concentrations of bitter gourd oil (100, 250, 500, 750, 1,000µg/mL), positive control, and negative control. The soaked discs were added to the Mueller-Hinton agar containing uniformly distributed *Streptococcus pyogenes *and incubated at 37°C for 48 hours (Figures 2, 3). The zone of inhibition was determined by measuring the clear zones around each disc.

Statistical analysis

Descriptive analysis was conducted, and mean ± standard deviation of the zone of inhibition for different concentrations of bitter gourd oil, positive control, and negative control was calculated. To ascertain the significant difference between the groups, analysis was conducted using the ANOVA test. A statistically significant result was defined as a p-value of less than 0.05.

## Results

Phytochemical analysis showed positive results for alkaloids, terpenoids, flavonoids, and phenols. A negative alkaline reagent test indicated the absence of quinones in the bitter gourd oil sample (Table 1).

**Table 1:** Phytochemical analysis (qualitative) of bitter gourd oil

Phytochemicals	Name of the test	Interpretation
Alkaloids	Mayer's test	+
Terpenoids	Salkowski test	+
Quinones	Alkaline reagent test	-
Flavonoids	Aluminum chloride test	+
Phenols	Ferric chloride test	+

**Figure 4:**
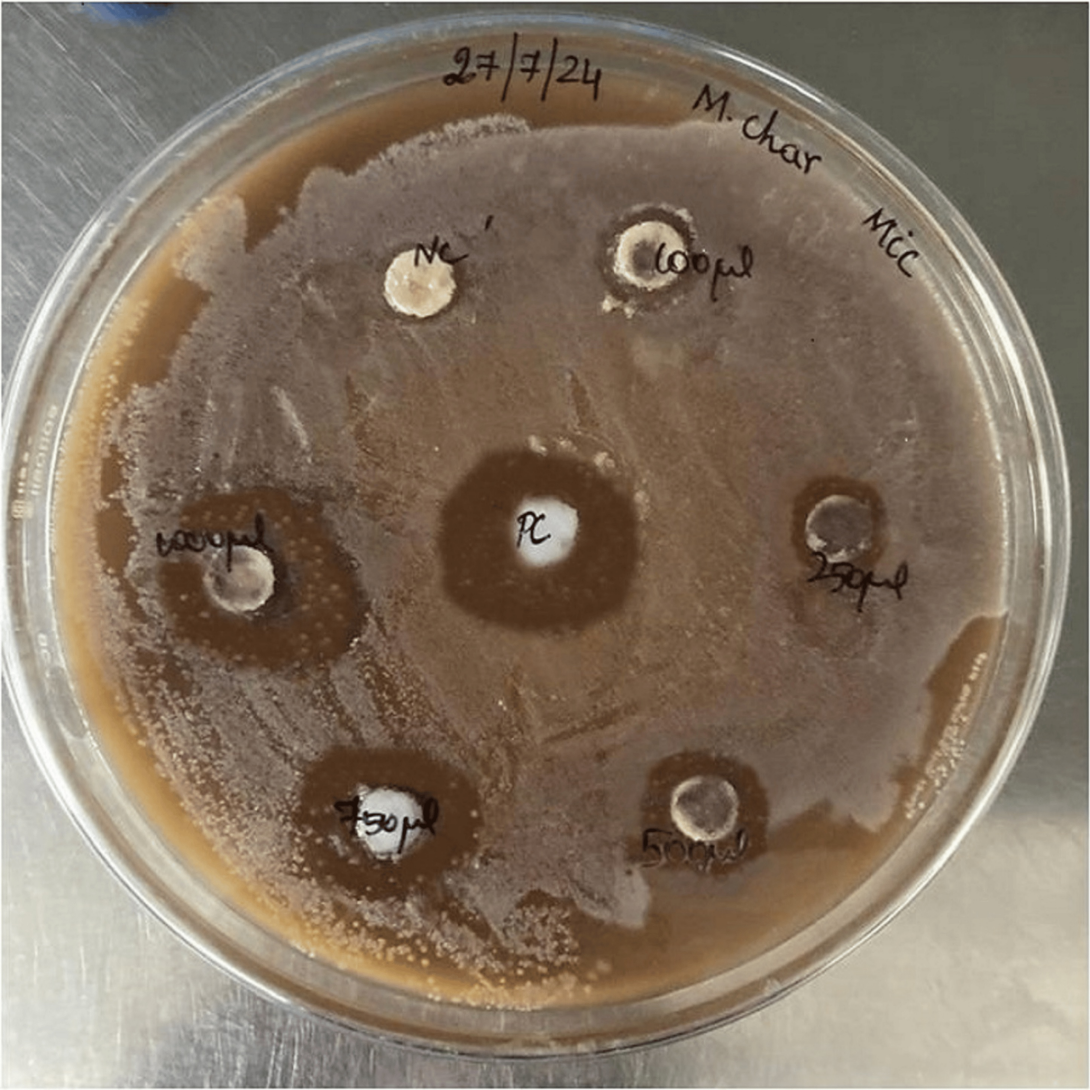
Minimum inhibitory concentration of Momordica charantia oil (after incubation)

The appearance of clear zones around each disc containing different concentrations of *Momordica charantia* oil explains the antibacterial activity exhibited by the oil extract (Figure 4). The bitter gourd oil showed a zone of inhibition measuring 6.4 ± 0.84mm at a concentration of 100µL. An inhibitory zone of 9.2 ± 0.91 was seen when the concentration was increased to 250µL. The inhibition zone extended to 12 ± 1.05mm at 500µL, indicating increasing antimicrobial activity. The biggest inhibition zone measured was 18 ± 1.56mm at the highest concentration of 1,000µL, whereas the concentration of 750µL yielded a mean inhibitory zone of 15 ± 0.81mm. In contrast, the zone of inhibition for amoxicillin (positive control) was 22 ± 0.66mm. Table 2 shows the MIC of bitter gourd oil against *Streptococcus pyogenes*. The average zone of inhibition exhibited by the highest concentration of *Momordica charantia*, positive control (amoxicillin 100µg/mL), and negative control (hexane 100µL) were compared using the ANOVA test (Table 3). Amoxicillin demonstrated a substantially greater zone of inhibition compared to *Momordica charantia *oil (1,000µg/mL), suggesting that bitter gourd can have a similar antibacterial action at higher concentrations (1,000µg/mL). Hexane (negative control) showed no zone of inhibition against the test organism. According to these findings, *Momordica charantia *oil has a strong antibacterial effect on *Streptococcus pyogenes*, with larger inhibition zones often resulting from higher concentrations.

**Table 2:** Minimum inhibitory concentration of bitter gourd oil against Streptococcus pyogenes A p-value of <0.05 is considered statistically significant

Concentration of bitter gourd oil	Zone of inhibition (in mm), mean ± standard deviation	Standard error	F	p-Value
100µg/mL	6.4 ± 0.84	0.26667	614.85	0.001
250µg/mL	9.2 ± 0.91	0.29059		
500µg/mL	12 ± 1.05	0.33333		
750µg/mL	15 ± 0.81	0.25820		
1,000µg/mL	18 ± 1.56	0.49441		
Amoxicillin 100µg/mL (positive control)	22 ± 0.66	0.21000		
Hexane 100µL (negative control)	0	0.00000		

**Table 3:** Comparison of Momordica charantia (bitter gourd oil) with positive and negative controls using the ANOVA test A p-value of <0.05 is considered statistically significant

Concentrations	Zone of inhibition (in mm), mean ± standard deviation	F	p-Value
*Momordica charantia* oil (1,000µg/mL)	18 ± 1.56	1426.15	0.001
Amoxicillin 100µg/mL (positive control)	22 ± 0.66		
Hexane 100µL (negative control)	0.0000		

## Discussion

The phytochemicals present in *Momordica charantia* oil exhibit antimicrobial action against a variety of microbial species. Trombetta et al. described that terpenoids can penetrate the bacterial cell membrane and interact with intracellular components, leading to antibacterial activity [10]. Flavonoids bind to the extracellular proteins and disrupt the bacterial cell walls. Peeran et al. conducted a phytochemical analysis to determine the various phytochemicals present in the bitter gourd extract. The study found that phenols, flavonoids, terpenoids, and alkaloids were present and were similar to the results of the present study [11]. Mada et al. reported in a study that phytochemicals such as flavonoids, phenolic compounds, and alkaloids were found to be present and contributed to the various medicinal properties of bitter gourd oil [12]. Significant antibacterial action has been shown by polysaccharides isolated from *Momordica charantia* against a variety of bacterial species such as *Staphylococcus aureus *and *Escherichia coli* [13].

Inhibitory zones can be divided into sensitive, intermediate, and resistant categories [14]. Inhibitory zones ≥17mm are considered to be sensitive, and zones 14-16mm and ≤13mm were categorized as intermediate and resistant. In this study, different concentrations of bitter gourd oil were compared to amoxicillin, which is a widely used broad-spectrum antibiotic to act against periodontal pathogens. In this study, amoxicillin produced a zone of inhibition of 22 ± 0.66, which is considered to be under the sensitive category. No zone of inhibition around hexane (negative control) containing disc revealed the absence of antibacterial properties of hexane.

Lower concentrations of bitter gourd oil such as 100µg/mL, 250µg/mL, and 500µg/mL exhibited an average MIC of 6.4 ± 0.84, 9.2 ± 0.91, and 12 ± 1,.05 respectively, and are included in the resistant category. Discs with 750µg/mL concentrations were categorized as intermediate, with an average inhibitory zone of 15 ± 0.81. An MIC of 18 ± 1.56 was exhibited by higher concentrations such as 1,000µg/mL, which was considered to be in the sensitive category. The results of the study revealed that the bitter gourd oil extracted from its seeds possesses antibacterial properties and that its activity increased with increasing concentration of the bitter gourd oil.

Chronic periodontitis is characterized by abnormal and exaggerated host immune and inflammatory response with marked elevation in proinflammatory cytokines and reactive oxygen species (ROS) in response to the toxins and enzymes released by the pathogenic microorganisms [15,16]. Bioactive compounds of *Momordica charantia* such as charantadiol A inhibit the production of proinflammatory cytokines such as interleukin-6, interleukin-8, and tumor necrosis factor-α [17]. Few studies demonstrated the antioxidant properties of *Momordica charantia* extracts and concluded that they can promote the wound-healing process of the diseased periodontium [18,19]. Owing to its antioxidant and anti-inflammatory properties, bitter gourd oil has an additional privilege of potential use in the treatment of chronic periodontitis in diabetic individuals.

Limitations of the study include limited sample size and evaluation of the antimicrobial action on only primary colonizing pathogenic bacteria. Studies evaluating gram-negative bacteria, which are keystone pathogens in the development of periodontal disease, would be warranted by additional research.

## Conclusions

The study demonstrated that higher concentrations (1,000µg/mL) of bitter gourd oil exhibit larger zones of inhibition similar to amoxicillin, which is one of the most commonly used antibiotics against periodontal microorganisms. Due to its availability and reduced risk of allergic reactions, *Momordica charantia *(Bitter gourd) may have the potential in the future to replace antimicrobial agents and can be considered a suitable option for treating periodontal disease.
